# Innate Immune Cytokine Profiling and Biomarker Identification for Outcome in Dengue Patients

**DOI:** 10.3389/fimmu.2021.677874

**Published:** 2021-07-14

**Authors:** Sai Pallavi Pradeep, Pooja Hoovina Venkatesh, Nageswar R. Manchala, Arjun Vayal Veedu, Rajani K. Basavaraju, Leela Selvasundari, Manikanta Ramakrishna, Yogitha Chandrakiran, Vishwanath Krishnamurthy, Shivaranjani Holigi, Tinku Thomas, Cecil R. Ross, Mary Dias, Vijaya Satchidanandam

**Affiliations:** ^1^ Department of Microbiology and Cell Biology, Indian Institute of Science, Bengaluru, India; ^2^ Division of Infectious Diseases Unit, St. John’s Research Institute, St. John’s Medical College, Bengaluru, India; ^3^ Department of Medicine, Kempegowda Institute of Medical Sciences and Research Centre, Bengaluru, India; ^4^ Department of Medicine, M S Ramaiah Medical College, Bengaluru, India; ^5^ Department of Medicine, Bengaluru Medical College and Research Institute, Bengaluru, India; ^6^ Department of Biostatistics, St. John’s Medical College, Bengaluru, India; ^7^ Department of Medicine, St. John’s Medical College, Bengaluru, India; ^8^ Department of Microbiology, St. John’s Medical College, Bengaluru, India

**Keywords:** dengue virus, innate immune cells, cytokines, biomarker, progression to severity, flow cytometry, intracellular cytokine staining (ICS)

## Abstract

**Background:**

Early biomarkers of progression to severe dengue are urgently required to enable effective patient management and control treatment costs. Innate immune cells, which comprise the earliest responders to infection and along with the cytokines and chemokines they secrete, play a vital role in orchestrating the subsequent adaptive immune response and have been implicated in the enhancement of infection and “cytokine storm” associated with dengue severity. We investigated the early innate immune cytokine profile of dengue patients during acute phase of disease in a prospective blinded study that included subjects with acute dengue and febrile controls from four major hospitals in Bengaluru, India along with healthy controls. We used intracellular cytokine staining and flow cytometry to identify innate immune biomarkers that can predict progression to severe dengue.

**Results:**

Dengue infection resulted in enhanced secretion of multiple cytokines by all queried innate immune cell subsets, dominated by TNF-α from CD56^+^CD3^+^ NKT cells, monocyte subsets, and granulocytes along with IFN-*γ* from CD56^+^CD3^+^ NKT cells. Of note, significantly higher proportions of TNF-α secreting granulocytes and monocyte subsets at admission were associated with mild dengue and minimal symptoms. Dengue NS1 antigenemia used as a surrogate of viral load directly correlated with proportion of cytokine-secreting innate immune cells and was significantly higher in those who went on to recover with minimal symptoms. In patients with secondary dengue or those with bleeding or elevated liver enzymes who revealed predisposition to severe outcomes, early activation as well as efficient downregulation of innate responses were compromised.

**Conclusion:**

Our findings suggested that faulty/delayed kinetics of innate immune activation and downregulation was a driver of disease severity. We identified IFN-*γ* ^+^CD56^+^CD3^+^ NKT cells and IL-6^+^ granulocytes at admission as novel early biomarkers that can predict the risk of progression to severity (composite AUC = 0.85–0.9). Strong correlations among multiple cytokine-secreting innate cell subsets revealed that coordinated early activation of the entire innate immune system in response to dengue virus infection contributed to resolution of infection and speedy recovery.

## Introduction

Dengue virus (DENV) afflicts ~130 million people annually with 70% contribution from Asia (1). Majority of patients present with dengue without warning signs (DwoWSs) while ~5–20% develop severe dengue (SD)—characterized by plasma leakage (dengue hemorrhagic fever; DHF) leading to shock (dengue shock syndrome; DSS) and/or organ impairment that predominantly manifest during defervescence (2, 3). While conventional wisdom holds that antibodies mitigate disease and aid pathogen clearance, in dengue the presence of heterotypic antibodies from prior exposure worsened disease severity (4). The phenomenon of antibody dependent enhancement (ADE) of viral infectivity of cultured cells by sera from severe dengue patients lent support to the epidemiological finding of enhanced risk for DHF/DSS in patients after a secondary dengue infection (5–7). However, multiple studies exist that reported both negative and positive correlations between dengue severity and dengue viral load, duration of viremia or NS1 antigen levels (8–17), suggesting that enhanced viral titers achieved by ADE alone cannot fully account for dengue severity. In addition to direct enhancement of uptake of antibody-bound virus leading to increased viral titers, excessive activation of innate immune cells by binding of dengue-specific antibodies to Fc receptors that are expressed generously on multiple innate cell subsets was also implicated in the ‘cytokine storm’ of vasoactive and pro-inflammatory cytokines. Monocytes that both support virus growth and express all three classes of Fc-receptors (Fc*γ*R, Fc*ϵ*R, and Fc*α*R) are likely major contributors (18, 19). The original antigenic sin theory attributes the severity observed during secondary infections to the activation of cross-reactive memory T cells generated during primary infection, which then secrete inflammatory cytokines and contribute to plasma leakage (20). Flow cytometry and transcriptome analyses of Indian and Thai dengue patients revealed CD8^+^ T-cell signaling deficits leading to a failure to secrete IFN-*γ* (21). NS3-stimulated T cells of SD patients from Sri Lanka failed to produce cytokines (22), which combined with spontaneous high production of multiple cytokines from unstimulated PBMCs (22), implicated innate cells as likely sources of the observed ‘cytokine storm’ in SD (23). This has been supported by *in vitro* assays of dengue virus-infected monocytes and natural killer (NK) cells (24, 25). Conflicting patterns for serum levels of TNF-α, IP-10, and IFN-*γ* as a function of severity were reported (26–29), perhaps due to variation in sampling time following infection. Cytokine measurements using enzyme-linked immunosorbent assay (ELISA) are incapable of identifying the source cell of the cytokines.

The need to identify SD cases early to facilitate interventions that reduce mortality has spawned numerous investigations to find biomarkers that can predict those most likely to progress to severity. We hypothesized that the early secretion of cytokines after onset of dengue symptoms would be contributed predominantly by innate immune cells that form the first line of defense against invading pathogens. Appropriately activated innate immune cells would contribute to early viral clearance and orchestrate the evolution of a host-protective adaptive immune response. Against this backdrop, in an effort to find newer biomarkers that define risk of progression to severity soon after dengue diagnosis and identify the source cells of inflammatory cytokines secreted early in dengue patients, we carried out a prospective blinded study to investigate the synthesis of cytokines by innate immune cells of dengue patients at the time of hospital presentation using intracellular cytokine staining and flow cytometry. Based on the extensive literature on serum cytokines in dengue patients that implicates these soluble mediators in protection/severity, we chose to query TNF-α, IP-10, IL-6, and IL-10 from monocytes and granulocytes along with TNF-α, IP-10, IL-10, and IFN-***γ*** from NK and NKT cell subsets.

## Materials and Methods

### Ethics Statement

This prospective study was carried out in accordance with the Declaration of Helsinki. Institutional ethics committee approval to conduct the study was obtained from the four participating hospitals: Bangalore Medical College and Research Institute (BMCRI; BMCRI/PS/25/2018-19), Kempegowda Institute of Medical Sciences (KIMS; KIMS/IEC/A1-2018), St. John’s Medical College (SJMC; IEC/1/473/2019), M S Ramaiah Medical College (RMCH; MSRMC/EC/19) and Indian Institute of Science (IISc; 10-14032018), Bengaluru, India.

### Study Subjects and Clinical Data Collection

**Figure 1:**
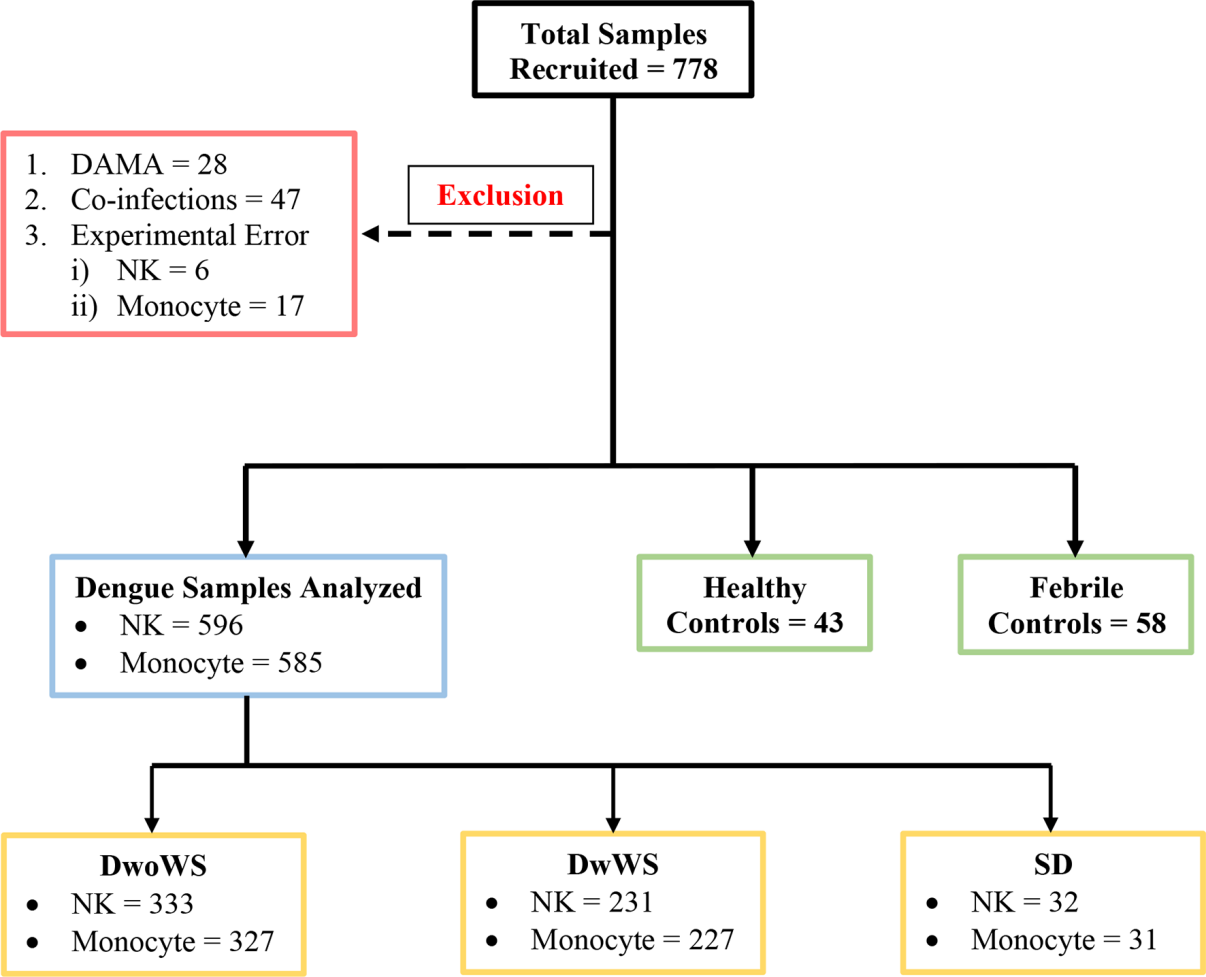
Schematic of patient recruitment. A total of 778 patients were initially recruited; patients who were discharged against medical advice (DAMA), those with co-infections (*i.e.* typhoid, sepsis, malaria, urinary tract infection, hepatitis B infection), and samples with experimental errors were excluded as shown. DwoWS, dengue without warning signs; DwWS, dengue with warning signs; SD, severe dengue; NK, natural killer cell.

For this prospective blinded study, 778 participants were enrolled at the fever clinics of the four hospitals listed above during the annual dengue season following onset of monsoon rains between June 24 and November 29, 2019 (**Figure 1**). Written informed consent was obtained from all participants before sample collection. Consecutive suspected dengue patients who tested positive and negative using a dengue specific NS1/IgM rapid dengue day 1 test kit (J Mitra and Co., India; https://jmitra.co.in/services_details.aspx?id=12&name=Dengue%20Day%201%20Test) were recruited as patients and febrile controls (FCs) respectively; both these fever groups were controlled by healthy volunteers with no illness for the past 3 months (HC; **Figure 1**). Of the patients, 166 tested positive for both dengue-specific NS1 and IgM while 167 and 263 tested positive for only IgM and only NS1, respectively. DENV infection in all these recruited volunteers was further confirmed using commercial IgM, IgG, and NS1 ELISA kits (Panbio, Brisbane, Australia) (3, 30, 31). Two hundred seventy seven patients tested NS1 positive following confirmatory tests. Blood samples were coded and labeled as DEN/hospital/19/### before being sent to IISc for flow cytometry investigations. Investigators who processed the blood samples for flow cytometry, acquired and analyzed the flow cytometry data were blinded to the identity of patients/controls. Raw and analyzed flow cytometry data were submitted to the clinical statistician who then revealed patient details by unblinding. Statistical analysis of all data reported in this manuscript was subsequently carried out.

Dengue without warning signs (DwoWSs) was defined by headache, body ache, rash, nausea, or mild bleeding, while dengue with warning signs (DwWSs) included symptoms like persistent vomiting, mucosal bleeding, pleural effusion, ascites, and hepatomegaly; severe dengue (SD) included symptoms such as plasma leakage, ≥1,000 IU/L of alanine aminotransferase/aspartate aminotransferase (ALT/AST), severe bleeding which leads to shock, and/or organ impairment, as defined by the World Health Organization (WHO) categorization of dengue severity (3). Demographic characteristics (*i.e.*, gender and age), clinical features (*i.e.*, days post symptom onset, nausea, headache, body ache, abdominal pain, rashes, splenomegaly, hepatomegaly, and bleeding manifestations), and routine hematological laboratory findings (*i.e.*, complete blood cell count, serum albumin, liver enzymes, platelet count, and hematocrit) were recorded. Patients were assigned bleed-scores (BSs) as follows: no bleeding, 0; petechiae, 1; epistaxis/gingival bleeding/menorrhagia, 2; gastrointestinal bleeding, 3; intracranial/intrapulmonary bleeding, 4. Plasma leakage in pleural and/or peritoneal cavities was confirmed using x-ray/ultrasound scans. HIV patients were not recruited. Data from samples of patients with co-infections (typhoid, sepsis, malaria, urinary tract infection, hepatitis B) or those who were discharged against medical advice (DAMA) and samples with experimental errors (clotted blood samples, QC failure of flow cytometer, sample processing errors) were excluded from analysis (**Figure 1**).

### Serology

DENV infection detected using the dengue specific NS1/IgM rapid dengue day 1 test kit (J Mitra and Co., India) was confirmed using commercial IgM, IgG, and NS1 ELISA kits (Panbio, Brisbane, Australia), and results were interpreted according to manufacturer’s instructions. The kits were used to distinguish primary (IgM to IgG ratio >1.2) from secondary (IgM to IgG ratio <1.2) infection (3, 30, 31). Primary dengue status was also assigned to those who tested positive for DENV specific NS1 (index value >1.1) but were negative for IgM and IgG.

### Dengue Serotyping

Dengue viral RNA detection and viral load determination were carried out using the Geno Sen’s^®^ DENGUE RG quantitative real time PCR kit (Genome Diagnostics Pvt. Ltd., New Delhi, India; Cat # 9111022) according to the manufacturer’s instructions. Dengue serotypes were determined using Geno Sen’s^®^ dengue typing 1/2/3/4 real time PCR kit (Genome Diagnostics Pvt. Ltd., New Delhi, India; Cat # 9111047) using a Qiagen Rotor-Gene Q instrument and software provided by the manufacturer.

### *Ex Vivo* Intracellular Cytokine Staining

Blood samples collected in sodium citrate vacutainer tubes (BD Biosciences, San Jose, California, USA) were immediately processed no later than 4 h from collection, and intracellular cytokine staining was carried out according to standard protocols (32–34). Briefly, RBCs from 500 µl blood were lysed using 4 ml of 1× ammonium chloride buffer (166 mM ammonium chloride, 9.9 mM potassium bicarbonate and 0.126 mM EDTA). The centrifuged cells were washed with 1× phosphate buffered saline (PBS) and stained with Fixable Viability Stain 450 [BD Biosciences, San Jose, California, USA; Cat#562247] for 10 min at room temperature, to exclude dead cells. This was followed by staining for appropriate surface markers (**Supplementary Table S1**) for 30 min at 4°C. The surface marker TLR-2 was superior to HLA-DR as a monocyte marker owing to its stable expression during infection in contrast to the latter which is reported to be downregulated in all manner of inflammatory conditions and was therefore used to identify monocytes (35). Cells were fixed with 2% paraformaldehyde [Sigma Aldrich, St Louis, Missouri, USA; Cat#P6148], washed, and permeabilized with 0.1% saponin. The permeabilized cells were stained with intracellular antibodies (monocyte panel: IL-6, IP-10, IL-10, TNF-α; NK panel: IP-10, IL-10, TNF-α, IFN-*γ*) for 30 min at 4°C (**Supplementary Table S1**). Cells were washed, resuspended in 1× PBS, and data were acquired on a Beckman Coulter DxFlex flow cytometer.

### Analysis of Flow Cytometry Data

**Figure 2:**
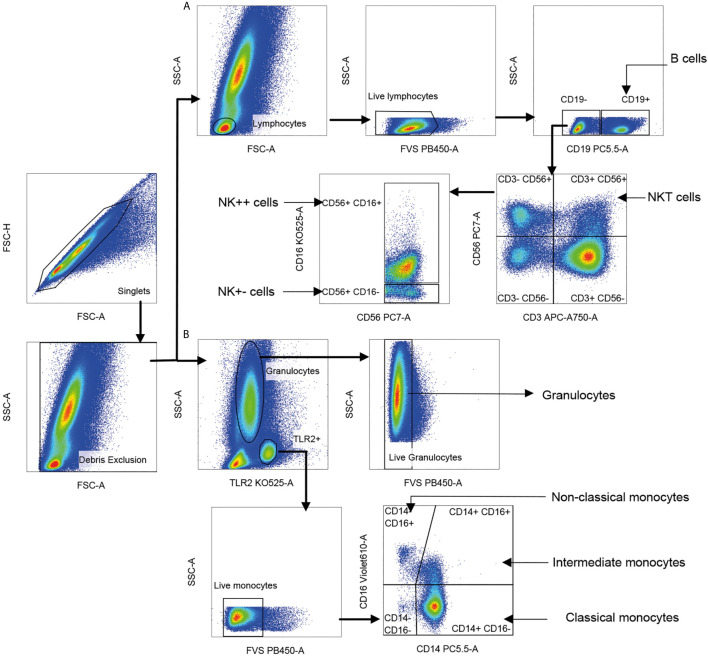
Gating strategy for innate immune cell subsets. Singlet cells were selected based on FSC-A and FSC-H scatter, and debris was excluded based on FSC-A and SSC-A. **(A)** Lymphocytes were gated using FSC-A and SSC-A scatter; dead cells and B cells staining for live/dead dye and CD19, respectively were sequentially excluded. Natural killer T (NKT) cells were identified as CD56^+^ CD3^+^ (CD56 *vs*. CD3). Two CD3-negative natural killer (NK) cell subsets were identified as CD56^+^ CD16^+^ (NK++) cells and CD56^+^ CD16^−^ (NK+−) cells displayed on CD56 *vs*. CD16. Cells positive for CD19 lineage marker were identified as B cells. **(B)** Live monocytes were identified as TLR-2^+^ and negative for live/dead dye. Three monocyte subsets were distinguished as CD14^+^CD16^−^ classical monocytes, CD14^+^CD16^+^ intermediate monocytes (IM) and CD14^−^CD16^+^ non-classical monocytes (NCM) based on CD16 *vs*. CD14. Live granulocytes were distinguished based on SSC-A scatter *vs*. TLR-2 followed by those negative for live/dead dye.

Gating strategy for NK, NKT, monocyte, and granulocyte subsets is shown in **Figure 2**. TLR-2 was used to define all monocytes (35). Monocyte subsets were defined based on CD14 and CD16. Total granulocytes were defined based on forward and side scatter while total NKT cells and NK cell subsets were defined based on expression of CD3, CD56, and CD16 (36). Percentages of various cell subsets in our cohort matched previously reported values (37–39). Cytokine-producing cells were represented as percentage of parent population. Control samples stained with surface antibodies alone were used to set the positive gates for each cytokine (**Supplementary Figure S1**). The number of events of each cytokine from each cell subset in comparison to the control is given in **Supplementary Table S2**. Data were analyzed using FlowJo software (version 10.6.1). Polyfunctional cytokine secretion was assessed by Boolean gating. The analyzed data from FlowJo were submitted to the clinical statistician for unblinding of patient characteristics prior to statistical analysis.

### Statistical Analysis

All analyses were done using IBM SPSS statistics 23.0 and GraphPad prism version 8. Significance between two or multiple groups was tested using Mann–Whitney *U* test (two-tailed) or non-parametric Kruskal–Wallis test with a Bonferroni correction for multiple comparisons, respectively. In patient cohort characteristics, normally distributed data were tested using one-way ANOVA. Chi square test of independence and Fisher’s exact test were used to evaluate the association of clinical parameters with WHO categorization of patients based on severity. Differences between proportions of primary and secondary infections across WHO categories were assessed using the Z test for proportions. Confidence intervals for odds ratio were determined using Baptista–Pike method. Spearman’s correlation (two-tailed) analysis was performed to assess the correlation if any between various cytokine-secreting cell subsets. Receiver operating characteristic (ROC) curve analysis was performed to assess accuracy of proposed biomarker, and 95% confidence intervals were calculated using Wilson/Brown method.

We performed multivariate binary logistic regression to compare DwoWS or DwWS/SD with worsened groups. Independent variables for multivariate analysis were selected if they were significantly different in univariate analysis (two-tailed Mann–Whitney *U* test for non-parametric continuous data and Chi square test for categorical variables). Required assumptions such as dichotomous mutually exclusive dependent variable, two or more independent variables, linear relationship between each independent variable and odds ratio, absence of multicollinearity were all met. We used a combination of monofunctional IL-6^+^ granulocytes with either total IFN-*γ* ^+^CD56^+^CD3^+^ NKT cells or IFN-*γ* ^+^TNF-α^+^CD56^+^CD3^+^ NKT cells to generate logistic regression models. The latter was not significant and was not used. Monofunctional IL-6^+^ granulocytes with total IFN-*γ* ^+^CD56^+^CD3^+^ NKT cell regression model were a good fit and confirmed by the Hosmer and Lemeshow goodness of fit test. The estimated probabilities obtained from logistic regression model were used to plot composite ROC curves.

## Results

### Cohort Characteristics

**Figure 3:**
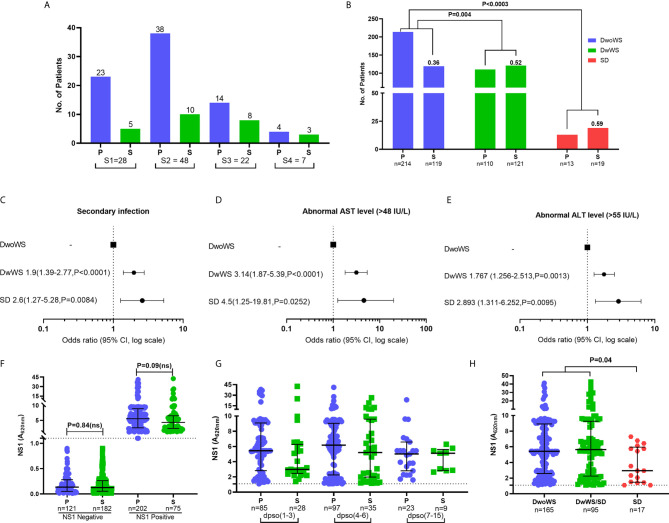
Clinical factors influencing severity in dengue patients. **(A)** Prevalence of dengue serotypes stratified based on serostatus. **(B)** Z-score test to compare the proportion of primary/secondary dengue in DwoWS, DwWS, and SD patients. Forest plot showing association of dengue severity with **(C)** serostatus **(D)** AST levels, **(E)** ALT levels and data are odds ratio (95% CI, P-value). **(F)** Positive and negative non-structural protein 1 (NS1) levels compared between primary and secondary patients. **(G)** All patients who had detectable dengue specific NS1 levels in serum were compared between primary and secondary based on days post symptom onset (dpso). **(H)** Levels of dengue NS1 compared between DwoWS, DwWS, and SD patients. P-values were determined using Kruskal–Wallis test, followed by Bonferroni correction for multiple comparisons between groups with median and IQR report. P, primary; S, secondary.

Of the 596 subjects with laboratory confirmed dengue who were included in the final data analyses (**Figure 1**), the mean age was 30.4 ± 10.79 (mean ± SD, range 17–69). They were admitted to the hospital at a median of 4 days (range 1–15) post symptom onset. Dengue specific IgM and IgG ELISA distinguished 336 (56.4%) primary from 256 (42.9%) secondary dengue patients. Two hundred seventy seven patients tested positive for dengue specific NS1. Of the 124 samples in which viral RNA was detectable, 121 were clearly categorized as primary (92) or secondary (29) while 3 were indeterminate. Of the 110 samples for which we obtained serotype data, DENV-2 was predominant (48) followed by serotypes 1 (28), 3 (22), and 4 (7) (**Figure 3A**). Three patients had co-infections of serotypes 2 and 4 while two had co-infections of serotypes 3 and 4. In our cohort, 333 (55.8%) patients were classified as DwoWS, 227 (38.7%) as DwWS, and 32 (5.4%) as SD. The clinical symptoms and laboratory parameters that correlated with the diagnoses are listed in **Table 1**. Our study design used the varying days of presentation at hospital to query the alterations in innate immune activation as a measure of kinetics of disease progression, since post admission, effects of medical interventions would influence subsequent alterations in innate immune status. Post recruitment and hospitalization, seven patients worsened sufficiently to transition from DwoWS to DwWS and one from DwWS to SD while two died. Interestingly, nine among 10 worsened patients were men.

**Table 1:** Demographic characteristics, clinical symptoms, and laboratory parameters of dengue patients.

Variable	DwoWS (n = 333)	DwWS (n = 231)	SD (n = 32)	P-value		
				DwoWS–DwWS	DwoWS–SD	DwWS–SD
Age; median (IQR)	26.8 (21.8, 35)	28 (22, 36.3)	29 (26.1, 43)	0.098^a^		
dpso; median (IQR)	4 (3,5)	4 (4,5)	4 (3,5)	**0.0008^a^**		
Male : Female	238:95	164:76	26:6	0.902^#^	0.238^#^	0.225^#^
Nausea; n (%)	127 (38.1)	127 (55)	18 (56.3)	**<0.0005** ^#^	** 0.046** ^#^	0.89^#^
Head ache; n (%)	155 (46.5)	132 (57.1)	16 (50)	**0.013** ^#^	0.708^#^	0.446^#^
Abdomen Pain; n (%)	0 (0)	109 (18.3)	16 (50)	**<0.0005** ^#^	<0.0005*	0.76^#^
Body Pain; n (%)	171 (51.4)	146 (63.2)	14 (43.8)	**0.005** ^#^	0.411^#^	0.035^#^
Rashes; n (%)	22(6.6)	25(10.8)	2(6.3)	**0.075** ^#^	1*	0.549*
Splenomegaly; n (%)	3(0.9)	8(3.5)	2(6.3)	0.03*	0.063*	0.349*
Hepatomegaly; n (%)	0	24(10.5)	4(12.5)	**<0.0005** ^#^	**<0.0005^a^**	0.536*
Petechiae; n (%)	1(0.3)	13(5.6)	0	**<0.0005** ^#^	1*	0.378*
Ascites; n (%)	0	39(16.9)	7(21.9)	**<0.0005** ^#^	<0.0005*	0.486^#^
Primary : Secondary	214:118	109:120	13:18	**<0.0005** ^#^	**0.008** ^#^	0.457^#^
Hemorrhage; n (%)	2 (0.6)	47 (20)	21 (65)	<0.0001*	<0.0001*	<0.0001*
Hemoglobin at enrollment; mean (SD)	14.43 (1.95)	14.48 (2.1)	15.14 (2.25)	0.10^b^		
Lowest platelet count (×10^3^); median (IQR)	50 (27,85)	29 (14-56)	16 (10,32)	**<0.0001**^a^		
Highest hematocrit %; mean (SD)	42.9 (5.23)	43.84 (5.5)	44.49 (6.4)	0.09^b^		
Albumin g/dl; median (IQR)	3.8 (3.5,4.1)	3.6 (3.3,4)	3.4 (3.2,3.7)	**<0.0001**^a^		
Aspartate transaminase IU/L; median (IQR)	86 (50,143)	114 (75,189)	123.5 (91,880)	**<0.0001**^a^		
Alanine transaminase IU/L; median (IQR)	56 (29.5,95.5)	70 (45, 118)	98 (51, 265)	**<0.0001**^a^		

Data are represented as total number with percentage unless otherwise indicated. Bold values indicate statistically significant P-values obtained using the following tests: ^a^Kruskal–Wallis test; ^b^ANOVA; ^#^Chi square test; *Fisher’s exact test. dpso, days post symptom onset; IQR, inter quartile range; n, number of patients; DwoWS, dengue without warning signs; DwWS, dengue with warning signs; SD, severe dengue.

In keeping with the extensive literature documenting the propensity for severe manifestations in secondary dengue, the odds of severity in our cohort were also greater in patients with secondary dengue (**Figures 3B, C**); also, patients with secondary dengue had high liver enzymes (**Figures 3D, E**). As in multiple reports that showed similar or greater viral titers in primary dengue patients (10, 16, 40, 41), levels of measurable NS1 antigenemia were similar in primary and secondary patients of our cohort (**Figure 3F**). These plasma NS1 levels did not vary as a function of disease duration and were comparable between primary and secondary dengue patients across all time frames (**Figure 3G**). Interestingly, we observed significantly reduced NS1 antigenemia in the 17 SD (eight primary and nine secondary) relative to DwoWS and DwWS patients (**Figure 3H**).

### DENV Activates Cytokine Production From Multiple Innate Immune Cells

**Figure 4:**
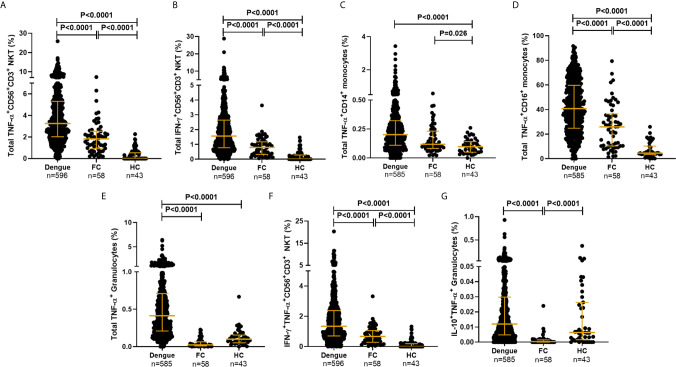
DENV activated cytokine secretion from innate immune cells. Frequency of total **(A)** TNF-α^+^CD56^+^CD3^+^ NKT cells, **(B)** IFN-*γ* ^+^CD56^+^CD3^+^NKT cells, **(C)** TNF-α^+^CD14^+^ monocytes, **(D)** TNF-α^+^CD16^+^ monocytes, **(E)** TNF-α^+^ granulocytes, **(F)** IFN-*γ* ^+^TNF-α^+^CD56^+^CD3^+^ NKT cells and **(G)** IL-10^+^TNF-α^+^ granulocytes from dengue patients compared to febrile controls (FC) and healthy controls (HC). Each dot represents one subject. P-value determined using Kruskal–Wallis test, followed by Bonferroni correction for multiple comparisons with median and IQR reported.

The gating strategy used to detect synthesis of the various cytokines by multiple innate immune cell subsets along with experimental controls is elaborated in **Figure 2** and **Supplementary Figure S1**. CD19 marker was used to gate out B cells and reliably identify CD56^+^CD3^+^ NKT and the two CD3^−^ NK cell subsets. The proportions of the various cell subsets in our samples were in conformation with those reported for these human immune cell subsets (39), further validating the antibody staining panels (**Supplementary Table S3**). We observed that dengue infection, in contrast to fevers from other etiologies, caused a surge in the production of inflammatory cytokines from a variety of innate immune cells (**Figures 4A–E**). TNF-α was by far the most abundant cytokine produced by multiple cell subsets from a vast majority of patients, while an impressive 95.5% of patients synthesized IFN-*γ* from CD56^+^CD3^+^ NKT cells (**Figure 4** and **Supplementary Table S4**). We also observed significantly greater numbers of dual-functional IL-10^+^TNF-α^+^ granulocytes and IFN-γ^+^TNF-α^+^CD56^+^CD3^+^ NKT cells in dengue patients than in febrile controls (**Figures 4F, G**), although the percentages of the former were low. Thus, in addition to monocytes which support dengue replication (24), all other innate cell subsets were also activated by DENV. Total TNF-α^+^ percentages within all subsets displayed strong positive correlation with one another (r = 0.63–0.77, P < 0.0001), while IFN-*γ* ^+^ and IFN-*γ* ^+^TNF-α^+^ within CD56^+^CD16^+^ NK or CD56^+^CD3^+^ NKT cells positively correlated with each other as expected, demonstrating synchronized activation of all innate cells by DENV. As reported previously (42), DENV infection significantly increased the numbers of CD14^+^ and CD14^+^CD16^+^ monocytes and significantly reduced CD56^+^CD3^+^ NKT cell numbers compared to controls (**Supplementary Table S3** and **Supplementary Figures S2A, B**). Some cell subsets, especially CD14^+^ monocytes, granulocytes, and CD19^+^ B cells progressively expanded with increase in disease severity (**Supplementary Table S3**). CD14^+^CD16^+^ intermediate monocytes were the highest per cell secretors of all cytokines (**Supplementary Figures S2C–G** and **Supplementary Table S4**). The median florescence intensity (MFI) was comparable across severity groups except for TNF-α^+^CD56^+^CD3^+^ NKT cells (**Supplementary Figures S2H, I**).

### Cytokine Synthesis by Innate Immune Cells Directly Correlates With Good Outcome

**Figure 5:**
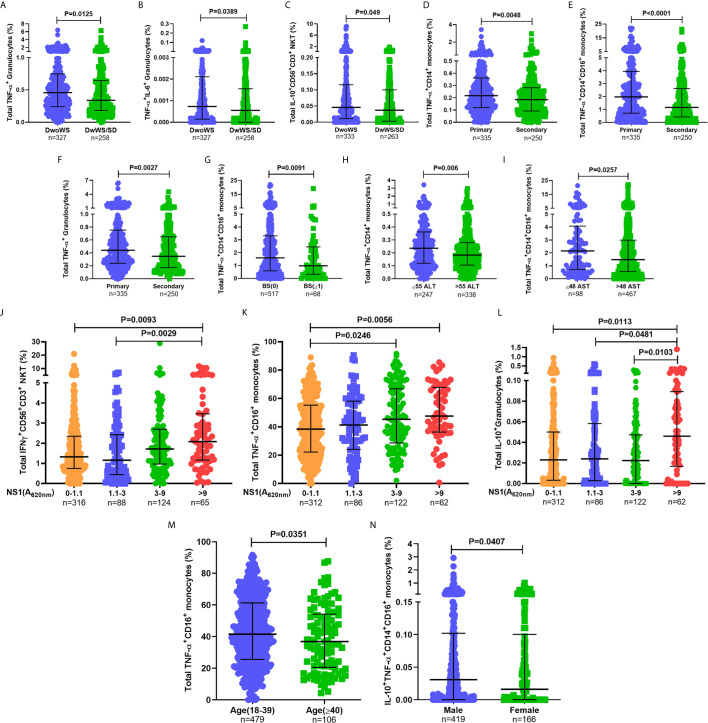
Higher percentages of innate immune cells secreting inflammatory cytokines were associated with better prognosis. Frequency of **(A)** total TNF-α^+^ granulocytes, **(B)** TNF-α^+^IL-6^+^ granulocytes, and **(C)** total IL-10^+^CD56^+^CD3^+^ NKT cells compared between DwoWS (circle) and DwWS/SD (square). Frequency of total **(D)** TNF-α^+^CD14^+^ monocytes, **(E)** TNF-α^+^CD14^+^CD16^+^ intermediate monocytes and **(F)** TNF-α^+^ granulocytes compared between primary (circle) and secondary patients (square). **(G)** Frequency of total TNF-α^+^CD14^+^CD16^+^ monocytes compared between bleed-scores 0 [BS(0); circle] and bleed-scores ≥1 (BS ≥ 1; square). Frequency of total **(H)** TNF-α^+^CD14^+^ monocytes and **(I)** TNF-α^+^CD14^+^CD16^+^ monocytes compared between normal (circle) and elevated (square) ALT (>55 IU/L; **E**) and AST (>48 IU/L; **F**), respectively. Frequency of total **(J)** IFN-*γ* ^+^CD56^+^CD3^+^ NKT cells, **(K)** TNF-α^+^CD16^+^ non-classical monocytes and **(L)** IL-10^+^ granulocytes compared between patients with undetectable NS1 (A620 nm 0–1.1), low NS1 (A620 nm 1.1 to 3), intermediate NS1 (A620 nm 3 to 9), and high NS1 (A620 nm> 9) values. **(M)** Frequency of total TNF-α^+^CD16^+^ non-classical monocytes in young (18–39 years; circle) compared to old (≥40 years; square) patients. **(N)** Frequency of IL-10^+^TNF-α^+^ CD14^+^CD16^+^ intermediate monocytes in men (circle) compared to women (square). P-values were determined using Mann–Whitney *U* test and Kruskal–Wallis test, followed by Bonferroni correction for multiple comparisons between groups. Median with IQR is reported.

When assessed as a function of disease severity, a significantly greater proportion of TNF-α^+^ granulocytes was evident in DwoWS relative to DwWS/SD (**Figure 5A**), a trend also evident for dual production of TNF-α and IL-6 by this cell subset and IL-10 production by CD56^+^CD3^+^ NKT cells (**Figures 5B, C**). It is to be noted that the percentages of the latter were low. Patients with secondary dengue who were predisposed to greater severity (**Figures 3B, C**) also displayed a significantly lower proportion of TNF-α producing monocytes and granulocytes (**Figures 5D–F**), suggesting that the reported (5, 6) deleterious role of pre-existing immunity on disease outcomes engenders sub-optimal innate immune activation. When we used bleed-scores or liver enzyme levels as surrogates of severity, those with no bleeding or with normal liver enzyme levels carried a significantly greater proportion of TNF-α producing monocyte subsets compared to those with varying degrees of hemorrhage or abnormal liver enzyme levels (**Figures 5G–I**). Higher NS1 levels, which are associated with milder forms of dengue as shown earlier **(** **Figure 3H**), also correlated directly with high levels of IFN-*γ* from CD56^+^CD3^+^ NKT cells, TNF-α from CD16^+^ monocytes and IL-10 from granulocytes (**Figures 5J–L**), suggesting a requirement for high viral antigen levels to achieve efficient innate cell activation.

In addition, we also observed age and gender related differences in cytokine production from monocyte subsets (**Figures 5M, N**). Despite these variations within the cohort based on gender, age, and primary/secondary dengue, data in **Figure 5** revealed that higher percentages of innate cell subsets synthesizing cytokines early during dengue infection impressively correlated with better prognosis as suggested by earlier blood transcriptome studies (43).

### Biomarker Performance of Cytokine-Producing Innate Immune Cell Subsets

**Figure 6:**
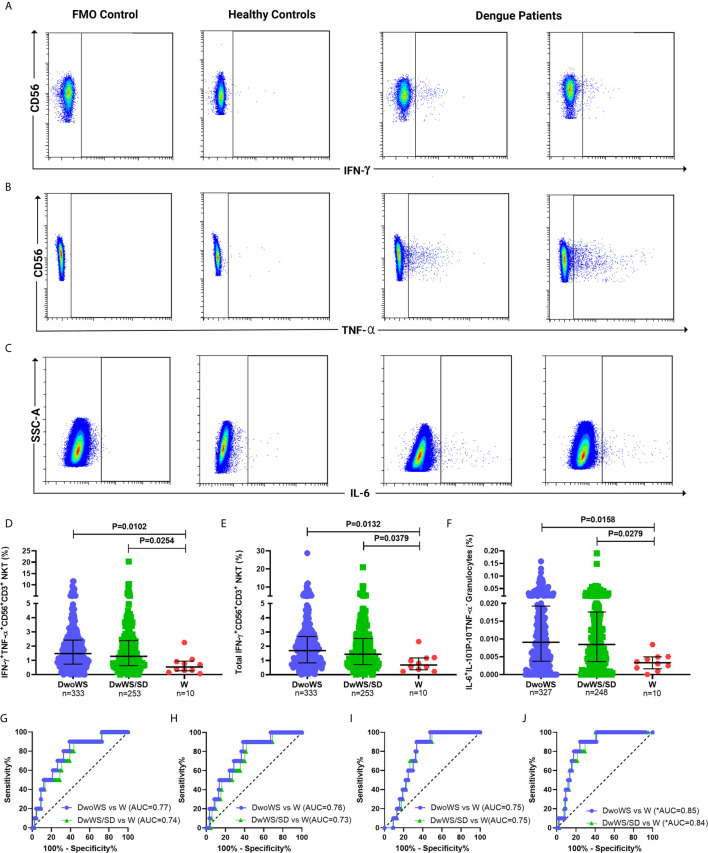
Innate cytokine-secreting cells predicted outcome in dengue patients. Pseudo color flow cytometry plots for representative patients displaying FMO control, healthy control, and dengue patient samples for secretion of **(A)** IFN-*γ* from CD56^+^CD3^+^NKT cells, **(B)** TNF-α from CD56^+^CD3^+^NKT cells, and **(C)** IL-6 from granulocytes. Frequency of **(D)** IFN-*γ* ^+^TNF-α^+^CD56^+^CD3^+^ NKT cells, **(E)** total IFN-*γ* ^+^CD56^+^CD3^+^ NKT cells and **(F)** monofunctional IL-6^+^ granulocytes compared between DwoWS (circle), DwWS/SD (square), and worsened (W; triangle) patients. P-value with median and IQR reported. ROC curve of **(G)** IFN-*γ* ^+^TNF-α^+^CD56^+^CD3^+^ NKT cells, **(H)** total IFN-*γ* ^+^CD56^+^CD3^+^ NKT cells **(I)** monofunctional IL-6^+^ granulocytes and **(J)** composite ROC curve for total IFN-*γ* ^+^CD56^+^CD3^+^ NKT cells combined with monofunctional IL-6^+^ granulocytes compared between DwoWS (purple) or DwWS/SD (green) and worsened patients. *AUC, composite AUC.

We wished to explore early cytokine-producing innate immune cells for potential biomarkers that can predict risk of progression of a patient to severity regardless of serostatus. We therefore compared all primary and secondary patients who worsened after recruitment (as evidenced by a shift from DwoWS/DwWS to DwWS/SD or death) as a single group, with patients who readily recovered from DwoWS and DwWS/SD. Recovered DwoWS and DwWS/SD patients both carried a significantly greater proportion of IFN-*γ* ^+^TNF-α^+^ and IFN-*γ* ^+^-CD56^+^CD3^+^NKT cells and monofunctional IL-6^+^ granulocytes (**Figures 6A–F**) relative to worsened patients. To assess the biomarker performance of these cell subsets, receiver operating characteristic (ROC) curve analysis was performed. IFN-*γ* ^+^TNF-α^+^CD56^+^CD3^+^, IFN-*γ^+^*CD56^+^CD3^+^ NKT and monofunctional IL-6^+^ granulocytes provided AUC of 0.77, 0.76, and 0.75 respectively with 90% sensitivity and 60 to 66% specificity when DwoWS was compared with the worsened group (**Figures 6G–I**). Since IFN-γ^+^TNF-α^+^CD56^+^CD3^+^ NKT cells and total IFN-*γ* ^+^CD56^+^CD3^+^ NKT cells directly correlated with each other as would be expected, we combined IL-6^+^ granulocytes and IFN-γ^+^CD56^+^CD3^+^ NKT cells using binary logistic regression to obtain a composite AUC of 0.85 (**Figure 6J** and **Table 2**). Analysis revealed that every one percentage rise in IFN-*γ* ^+^CD56^+^CD3^+^ NKT cells resulted in 3.12-fold lower odds of worsening (95% CI=1.1–9.2, P = 0.035). In patients with elevated AST, this composite biomarker predicted the progression to severity with higher accuracy (AUC = 0.9) and displayed 100% sensitivity with 81.9% specificity (**Table 2**). We observed that although liver enzymes were able to distinguish between DwoWS and SD (**Figures 3D, E**) as also reported earlier (44), they could not predict the risk of progression to severity (data not shown).

**Table 2:** Biomarker performance of IFN-*γ* ^+^CD56^+^CD3^+^ NKT cells, IFN-*γ* ^+^TNF-α^+^CD56^+^CD3^+^ NKT cells and monofunctional IL-6^+^ granulocytes using ROC analysis in total and homogeneous patient groups.

Cell subset	Patient groups	AUC (95% CI)	Cut-off	Sensitivity (%)	Specificity (%)	Likelihood Ratio
**IFN-*γ*^+^CD56^+^CD3^+^ NKT cells**	Total	0.76 (0.64–0.88)	<1.202^a^	90	61.56	2.34
	Abnormal liver enzymes	0.83 (0.74–0.92)	<1.216^a^	100	60.32	2.52
**IFN-*γ*^+^TNF-α^+^ CD56^+^CD3^+^ NKT cells**	Total	0.77 (0.64–0.90)	<1.072^a^	90	60.96	2.31
	Abnormal liver enzymes	0.83 (0.74–0.93)	<1.072^a^	100	59.52	2.47
**IL-6^+^ granulocytes**	Total	0.75 (0.67–0.83)	<0.005^a^	90	66.36	2.67
**IL-6^+^ granulocytes, IFN-*γ*^+^CD56^+^CD3^+^ NKT cells**	Total	0.85 (0.78–0.92)	>0.042^b^	90	75.93	3.73
	Abnormal AST	0.90 (0.85–0.95)	>0.056^b^	100	81.9	5.53
	Abnormal liver enzymes	0.88 (0.83–0.94)	>0.51^b^	100	75.7	4.12

ROC analysis compared recovered DwoWS patients with those who worsened during the study.

AU, area under the curve; CI, confidence interval; ROC, receiver operating characteristic; DwoWS, dengue without warning signs. Cut-off represents (^a^) % cytokine-secreting cells or (^b^) probability obtained from regression analysis.

### Kinetics of Cytokine Production by Innate Immune Cells in Dengue Patients

**Figure 7:**
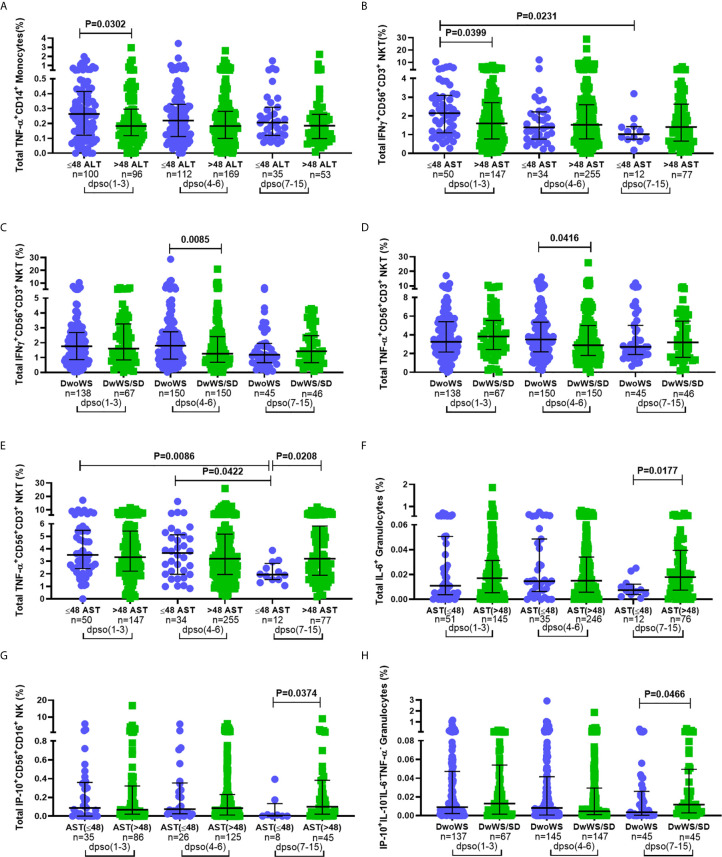
Correlation between disease severity and kinetics of innate immune cell activation. Frequency of total **(A)** TNF-α^+^CD14^+^ monocytes and **(B)** IFN-*γ* ^+^CD56^+^CD3^+^ NKT cells compared between normal (circle) and high (square) levels of ALT and AST (**A** and **B** respectively; IU/L). Frequency of total **(C)** IFN-*γ* ^+^CD56^+^CD3^+^ NKT cells and **(D)** TNF-α^+^CD56^+^CD3^+^ NKT compared between DwoWS (circle) and DwWS/SD (square). Frequency of total **(E)** TNF-α^+^CD56^+^CD3^+^ NKT cells **(F)** IL-6^+^ granulocytes in total cohort and **(G)** IP-10^+^CD56^+^CD16^+^ NK cells in primary dengue cohort compared between normal (circle) and high (square) AST levels. Frequency of **(H)** monofunctional IP-10^+^ granulocytes compared between DwoWS (circle) and DwWS/SD (square) severity groups. All comparisons were also carried out between time windows of hospital presentation during 1–3, 4-6 or 7–15 days post symptom onset (dpso). P-values were determined using Mann–Whitney *U* test between normal and abnormal levels of AST/ALT or DwoWS and DwWS/SD groups for any single time interval and Kruskal–Wallis test, followed by Bonferroni correction for multiple comparison of normal or abnormal AST or DwoWS or DwWS/SD patients between the three time intervals. Medians with IQR are reported.

Innate immune responses typically arise and attain peak levels early following infection. In order to query a link, if any, between kinetics of innate immune activation by DENV and disease severity, we compared innate cell cytokine production between different measures of severity at early (days 1–3), intermediate (days 4–6) and late (days 7–15) times of hospital presentation. Patients admitted 1–3 days post symptom onset (dpso) had significantly higher TNF-α and IFN-*γ* (**Figures 7A, B**)-synthesizing innate cells in those with normal compared to above-normal liver enzyme levels. Those admitted 4–6 dpso had a significantly greater proportion of IFN-*γ* ^+^CD56^+^CD3^+^ NKT cells as well as TNF-α-secreting CD56^+^CD3^+^ NKT cells in DwoWS relative to DwWS/SD (**Figures 7C, D**). In contrast to patients with normal liver enzyme levels, those with abnormally high levels failed to downregulate synthesis of TNF-α, IL-6, and IP-10 from different innate cell subsets during the late stage (7–15 dpso; **Figures 7E–G**). Severe dengue was also characterized by a late surge of IP-10 from granulocytes (**Figure 7H**). Thus, efficient attenuation of all innate cytokines during later stages of dengue disease was just as important as robust early activation of cytokine synthesis by innate cells to avert severity.

## Discussion

Despite the demonstration of enhancement of viral infectivity in dengue by pre-existing immunity and the strong association of severity with high viral loads in multiple studies (9, 11, 15, 16, 45), other studies reporting no association between viral load and severity (8, 10, 13, 14) precluded its use as a biomarker to predict severity. While studies on cohorts from Thailand and Cuba have both demonstrated the highest proportion of severe cases in secondary dengue patients infected with serotype 2 following serotype 1 (46, 47), determining the serotype of both current and previous infection(s) requires technically skilled serology investigations that are not routinely used in diagnostic laboratories. Additionally, the virulence of circulating strains has been seen to decide severe clinical presentations (46). Most importantly, the frequently observed onset of severity during defervescence implicates host immunopathology in dengue severity along with viral determinants. Hence, we sought to identify easily measurable newer biomarkers based on early host innate immune responses to identify patients most likely to progress to severe states. The majority (56%) of our cohort being primary dengue patients suggested moderate endemicity and viral transmission, permitting analysis of innate immune responses with minimal interference from ‘multiplicity of confounding immunity patterns’ observed in hyper-endemic cohorts (46, 48). In our large cohort, secondary dengue increased the odds of severity, confirming the pathogenic role of preexisting immunity in dengue. As expected, young patients had better outcomes than those older. Unexpectedly, despite the significantly higher percentages of innate cells synthesizing cytokines in men compared to women, they did not confer an advantage in terms of outcomes; men dominated those who worsened post dengue diagnosis and admission, reminiscent of significantly more severe SARS-CoV-2 disease in men compared to women (49, 50).

This study, which queried the cellular source of innate inflammatory cytokines in a large, blinded dengue cohort, conclusively demonstrated the beneficial role of early innate immune cell activation in avoiding severe symptoms and ensuring recovery from dengue. The broad-based activation of the innate immune system resulting in mono and polyfunctional cytokine production by DENV convincingly correlated with good outcome. Particularly striking was the association of higher percentages of IL-10 secreting NKT cells with better outcomes despite their low percentages (**Figure 5C**). Seen along with the reported higher levels of serum IL-10 in severe dengue as well as the deleterious role of IL-10 in causing T cell apoptosis in acute severe dengue cases reported earlier from Sri Lanka (22, 51, 52), our findings suggest that early production of IL-10 from innate cells may contribute to preventing severe disease. Significantly higher innate activation associating with high serum NS1 levels used as a surrogate of viral load also suggested that high viral antigen levels early in infection contributed to vigorous stimulation of all innate cell subsets. As mentioned above, the relationship between dengue viral load and disease severity is rather tenuous with reports showing both positive and negative correlations (8, 10, 13, 14, 49, 50). In our large cohort however, the direct correlation between high NS1 antigenemia and good prognosis regardless of day post symptom onset was strong and convincing. The innate immune cytokine signature for each pathogen may be unique and rewarding to investigate.

We were also successful in studying cytokine production by granulocytes which are short lived and challenging to stain for intracellular markers. Indeed, granulocytes were the highest per cell secretors of IL-10 and the second highest per cell producers of TNF-α, IP-10, and IL-6, the last mentioned proving to be one of the biomarkers that effectively predicted risk of progression to severity. In SARS-CoV-2 patients the ratio of neutrophils to lymphocytes (NLR) was found to predict both mortality and severe manifestations (53–56). In our dengue cohort however, we observed no alterations in granulocyte to lymphocyte ratio between severity groups. An earlier report also demonstrated synthesis of TNF-α, IL-6, and IL-10 from circulating B cells, monocytes, and mDC in PBMC of pediatric dengue patients; culture supernatants from these purified B cells activated allogeneic T cells (57). This study in a small cohort however, did not identify biomarkers of severity.

Our analysis of the kinetics of innate cell activation provided multiple insights regarding the likely mechanisms underlying risks of progression to severity. Early activation of cytokine secretion from all innate cell subsets was significantly lower in those who manifested severe symptoms. Weaker activation of early innate effector mechanisms was also reported in dengue patients who later developed severe manifestations (58). Our observed persistence of innate cell-derived cytokines, especially TNF-α, IL-6, and IP-10 during late phase of disease which was in keeping with the reported slow resolution of serum inflammatory mediators in severe dengue patients (59), pointed to a contribution for dysregulated innate responses towards severity. Failure to downregulate serum IP-10 levels during convalescence earlier reported in dengue patients with warning signs in contrast to those without warning signs (29) is in keeping with our findings. Severe COVID-19 patients also displayed sustained plasma levels of IFN-α, IP-10, and IL-6 (60, 61); increased plasma IP-10 also correlated with liver impairment in HIV/HBV patients (62). In light of the reported requirement of IP-10 for B cell activation (63), abnormal high IP-10 levels during late stages of disease in severe dengue patients (**Figures 7G, H**) suggest a possible mechanism for the contribution of B cell mediated immunopathology to severe dengue (20). A potential role for persistently elevated innate responses in preventing the onset of adaptive immunity and provoking the reported defective TCR signaling and T cell apoptosis in SD (21, 22) deserves further investigation. Thus, failure to achieve both robust early activation and prompt attenuation of innate immune cells was a hallmark of severe dengue. The reported impaired innate immune activation in severe SARS-CoV-2 patients (60, 64) suggests that severe disease in multiple viral infections may be a shared consequence of defective modulation of kinetics of early host innate activation and its subsequent attenuation.

Our findings are in line with the 20 gene transcript signatures that included anti-viral IFN-*γ* signaling pathway genes being under-expressed in NK and NKT cells of SD patients (65). Invariant natural killer T (iNKT) cells have been reported to expand in acute dengue infections (66). An earlier study also reported higher percentages of IFN-*γ* secreting alpha galactosyl ceramide-stimulated iNKT cells in mild dengue patients despite greater percentages of CD69 positive activated iNKT cells in severe dengue patients (67). Earlier reports of biomarkers of dengue severity included circulating cytokines/chemokines measured by ELISA or were based on proteomics of serum or transcript profiling of immune cells (65, 68–71). Our composite biomarker relies on flow cytometry of fresh whole blood which is amenable for rapid reporting of results even from individual samples. Our composite biomarker comprising IFN-*γ* ^+^CD56^+^CD3^+^ NKT cells and IL-6^+^ granulocytes performed well despite limited number of patients with transitions to greater severity post recruitment. We included patients with both primary and secondary dengue and those with varying disease duration as a single group for the purpose of biomarker identification, in order to mimic and render it suitable for real life clinical settings. Potential enhancement of its performance by including additional hitherto unidentified cytokine-producing cells or plasma cytokines will further improve its utility. While biomarkers reliant on expensive instruments and high-end technical skills (65, 68, 69) may have limited utility in resource constrained geographies, ease of processing and ready availability of flow cytometers in diagnostic laboratories worldwide assure feasibility of host blood-based biomarker deployment.

## Data Availability Statement

The original contributions presented in the study are included in the article/**Supplementary Material**. Further inquiries can be directed to the corresponding author.

## Ethics Statement

The studies involving human participants were reviewed and approved by Bangalore Medical College and Research Institute (BMCRI; BMCRI/PS/25/2018-19), Kempegowda Institute of Medical Sciences (KIMS; KIMS/IEC/A1-2018), St. John’s Medical College (SJMC; IEC/1/473/2019), M S Ramaiah Medical College (RMCH; MSRMC/EC/19) and Indian Institute of Science (IISc; 10-14032018). The patients/participants provided their written informed consent to participate in this study.

## Author Contributions 

VS conceived, designed, planned and supervised the study, interpreted data and wrote the manuscript. SP and PH conducted the experiments, analyzed data and wrote the manuscript. TT oversaw the unblinding followed by statistical analysis of data. MD oversaw diagnostic tests and serotyping. CR, MD, VK, YC, SH, NM, AV, LS, RB, and MR were responsible for consenting and recruitment of patients, clinical sample collection and maintenance of case report forms. All authors contributed to the article and approved the submitted version.

## Funding

This work was funded by Rajiv Gandhi University of Health Sciences (Grant number – RGU/ADV.RES/016/2017-2018). The funding agency had no role in the study. Funds to cover the open access publication fees will be sourced from other grants.

## Conflict of Interest

The authors declare that the research was conducted in the absence of any commercial or financial relationships that could be construed as a potential conflict of interest.
