# Supplementary material for: Innate Immune Cytokine Profiling and Biomarker Identification for Outcome in Dengue Patients
Source: Front Immunol. 2021 Jul 14;12:677874. doi: 10.3389/fimmu.2021.677874 (PMC8318829; doi:10.3389/fimmu.2021.677874)
Supplement: Supplementary file 1 [file DataSheet_1.docx]

Innate immune cytokine profiling and biomarker identification for outcome in dengue patients

| **Title** | **Full Name** | **Last Name** |
| --- | --- | --- |
| Ms. | Sai Pallavi | Pradeep |
| Ms. | Pooja | Hoovina Venkatesh |
| Dr | Nageswar | R. Manchala |
| Mr. | Arjun | Vayal Veedu |
| Ms. | Rajani | K. Basavaraju |
| Ms. | Leela | Selvasundari |
| Mr. | Manikanta | Ramakrishna |
| Dr | Yogitha | Chandrakiran |
| Dr | Vishwanath | Krishnamurthy |
| Dr | Shivaranjani | Holigi |
| Dr | Tinku | Thomas |
| Dr | Cecil | R. Ross |
| Dr | Mary | Dias |
| **Corresponding Author** | | |
| Dr | Vijaya | Satchidanandam |

**Figure Legends**

**Supplementary Figure 1**. **Controls for flow cytometry**. Pseudo color flow cytometry plots for a representative patient comparing the FMO control (top row) with completely stained sample (bottom) for secretion of (**A**) TNF-α, (**B**) IP-10, (**C**) IL-10, (**D**) IL-6 and (**E**) IFN-γ from the indicated cell subsets. Abbreviations: CM - CD14^+^CD16^-^ classical monocytes; IM - CD14^+^CD16^+^ intermediate monocytes; NCM - CD14^-^CD16^+^ non-classical monocytes; G - Granulocytes; NKT - CD56^+^CD3^+^ natural killer T cells; NK^++^ - CD56^+^CD16^+^ natural killer cells; NK^+-^ - CD56^+^CD16^-^ natural killer cells.

**Supplementary Figure 2**. **Modulation of innate immune cell subsets by dengue infection and per cell secretion of cytokines from each cell subset**. Pseudo color flow cytometry plots for a representative patient and healthy control show (**A**) reduction in percentage of CD56^+^CD3^+^ NKT cells and (**B**) expansion of percentage of CD14^+^CD16^+^ intermediate monocytes in dengue patients relative to healthy control. MFI values for each cytokine were compared between subsets. (**C**) TNF-α (**D**) IP-10 (**E**) IL-10 (**F**) IFN-γ (**G**) IL-6 compared among cell subsets. P values were determined using Kruskal-Wallis test, followed by Bonferroni correction for multiple comparison between groups with median and IQR reported. **** P<0.0001. MFI of (**H**) total IFN-γ^+^ and (**I**) total TNF-α^+^ cells within CD56^+^CD3^+^ NKT cells compared between DwoWS and DwWS/SD. Mann-Whitney U test was performed and median with IQR are reported. Abbreviations: NK - CD56^+^ cells; NKT - CD56^+^CD3^+^ cells; T - CD3^+^ T cells; NCM - CD14^-^CD16^+^ non-classical monocytes; IM - CD14^+^CD16^+^ intermediate monocytes; CM - CD14^+^CD16^-^ classical monocytes; G – Granulocytes; NK^++^ - CD56^+^CD16^+^ natural killer cells; NK^+-^ - CD56^+^CD16^-^ natural killer cells; MFI - median florescence intensity.

**Supplementary Table 1. Antibodies used for flow cytometry.**

| **Target (Clone)** | **Species** | **Fluorochrome/Conjugate** | **Company** | **Catalog no.** |
| --- | --- | --- | --- | --- |
| **NK cell panel – Surface Markers** | | | | |
| CD19 (HIB19) | Mouse IgG1, κ | PE-Cy5 | BioLegend | 302210 |
| CD56 (B159) | Mouse IgG1, κ | PE-Cy7 | BD Bioscience | 557747 |
| CD3 (SK7) | Mouse BALB/cIgG1, κ | APC-H7 | BD Bioscience | 560176 |
| CD16 (3G8) | Mouse CDF1 IgG1, κ | BV510 | BD Bioscience | 563830 |
| **Monocyte Panel – Surface Markers** | | | | |
| CD14 (M5E2) | Mouse IgG2a, κ | PerCP- Cy5·5 | BD Bioscience | 550787 |
| TLR2 (11G7) | Mouse IgG1, κ | BV510 | BD Bioscience | 742767 |
| CD16 (3G8) | Mouse CDF1 IgG1, κ | BV605 | BD Bioscience | 563172 |
| **Intracellular Cytokine Markers** | | | | |
| IP-10 (6D4/D6/G2) | Mouse IgG2a, κ | PE | BD Bioscience | 555049 |
| IL-10 (JES3-19F1) | Rat IgG2a, κ | APC | BD Bioscience | 554707 |
| IFN-γ (B27) | Mouse IgG1, κ | BV605 | BD Bioscience | 562974 |
| TNF-α (MAb11) | Mouse IgG1, κ | BV750 | BD Bioscience | 566359 |
| IL-6 (MQ2-6A32) | Rat IgG2a, κ | FITC | BD Bioscience | 557696 |

**Supplementary Table 2**. **Total number of cytokine-secreting innate immune cell events from control and test samples.**

| **Cell Subsets** | **Events** | **TNF-α** | | **IL-6** | | **IP-10** | | **IL-10** | |
| --- | --- | --- | --- | --- | --- | --- | --- | --- | --- |
|  |  | ***Control*** | ***Test*** | ***Control*** | ***Test*** | ***Control*** | ***Test*** | ***Control*** | ***Test*** |
| **CM** | Median | 4 | 70 | 5 | 9 | 5 | 19 | 10 | 17 |
|  | IQR | 2-8 | 33-128 | 2-17 | 4-28 | 1-20 | 4-78 | 3-35 | 7-44 |
| **IM** | Median | 37 | 191 | 15 | 21 | 18 | 24 | 31 | 37 |
|  | IQR | 16-89 | 86-341 | 8-29 | 9-45 | 8-33 | 12-47 | 17-65 | 16-79 |
| **NCM** | Median | 2 | 564 | 2 | 3 | 1 | 4 | 4 | 6 |
|  | IQR | 1-4 | 324-927 | 0-5 | 1-8 | 0-4 | 1-9 | 1-10 | 2-15 |
| **Granulocytes** | Median | 38 | 1272 | 21 | 76 | 23 | 91 | 92 | 180 |
|  | IQR | 23-66 | 637-2136 | 9-47 | 40-138 | 11-49 | 40-223 | 56-147 | 110-268 |
|  |  | **TNF-α** | | **IFN-γ** | | **IP-10** | | **IL-10** | |
| **NKT** | Median | 6 | 364 | 4 | 160 | 2 | 12 | 4 | 11 |
|  | IQR | 3-13 | 186-665 | 1-8 | 81-310 | 0-7 | 4-35 | 1-9 | 4-23 |
| **NK++** | Median | 1 | 131 | 3 | 44 | 2 | 13 | 2 | 4 |
|  | IQR | 0-2 | 63-253 | 1-8 | 22-86 | 0-5 | 4-47 | 0-6 | 1-10 |
| **NK+-** | Median | 0 | 12 | 0 | 4 | 0 | 2 | 0 | 1 |
|  | IQR | 0-1 | 5-25 | 0-0 | 1-10 | 0-1 | 0-9 | 0-1 | 0-2 |

Abbreviations: IQR – Inter Quartile Range; CM – CD14^+^ CD16^-^ classical monocytes; IM – CD14^+^CD16^+^ intermediate monocytes; NCM – CD14^-^CD16^+^ non-classical monocytes NKT – CD56^+^CD3^+^ natural killer T cells; NK^++^ - CD56^+^CD16^+^ natural killer cells; NK^+-^ - CD56^+^CD16^-^ natural killer cells.

**Supplementary Table 3. Immune cell subsets in dengue, control groups and as a function of dengue severity.**

| **Cell Subsets** | **Dengue (n=596)** | | **FC (n=58)** | | **HC (n=43)** | | **P value** | |
| --- | --- | --- | --- | --- | --- | --- | --- | --- |
|  | ***%*** | ***Absolute count (/µl)*** | ***%*** | ***Absolute count (/µl)*** | ***%*** | ***Absolute count (/µl)*** | ***%*** | ***Absolute count (/µl)*** |
| **Lymphocytes** | 31.9 (21.9–39.7) | 1423 (923–2422) | 29.3 (20.8–41.1) | 1758 (849–2940) | 34.7 (22.8–40.4) | 1568 (737–2867) | ns^#^ | ns^#^ |
| **NK^++^ cells^a^** | 6.4 (4.2–9.0) | 92 (47–166) | 5.7 (3.8–8.8) | 106 (56–179) | 6.8 (4.3–10.4) | 105 (54–150) | ns^#^ | ns^#^ |
| **NK^+-^ cells^a^** | 1.1 (0.8–1.6) | 18 (9–31) | 0.9 (0.7–1.3) | 17 (8–29) | 0.7 (0.4–0.9) | 11 (4–19) | **<0.0001**^#^ | **0.0009**^#^ |
| **NKT cells^a^** | 4.9 (3.7–7.3) | 77 (45–132) | 6.9 (4.7–9.3) | 126 (67–208) | 6.7 (4.8–8.2) | 120 (33–186) | **<0.0001**^#^ | **0.0007**^#^ |
| **B cells^a^** | 10.6 (7.6–14.5) | 153 (81–305) | 8.6 (5.3–11.8) | 131 (62–241) | 8.7 (6.3–13.6) | 123 (58–280) | **0.0004**^#^ | ns^#^ |
| **T cells^a^** | 59.4 (52.8–64.9) | 815 (448–1373) | 57 (51.2–65.8) | 1013 (451–1503) | 54.3 (49–60.5) | 815 (382–1567) | **0.0250**^#^ | ns^#^ |
|  | **Dengue (n=585)** | | **FC (n=58)** | | **HC (n=43)** | |  |  |
| **Monocytes** | 6.4 (4.7–8.5) | 301 (173–464) | 5.2 (2.9–6.5) | 252 (149–357) | 4.3 (3.4–5.4) | 179 (113–296) | **<0.0001**^#^ | **0.0002**^#^ |
| **CM^b^** | 68.6 (60.6–76.2) | 228 (122–347) | 64.9 (53.3–73.9) | 178 (104–288) | 70.7 (64.9–74.2) | 140 (89–238) | **0.0229**^#^ | **0.0018**^#^ |
| **IM^b^** | 17.3 (12.6–24.7) | 53 (31–92) | 17.1 (13.6–22.2) | 44 (27–91) | 9.5 (7.7–11.9) | 20 (12–35) | **<0.0001**^#^ | **<0.0001**^#^ |
| **NCM^b^** | 3.2 (2.1–4.9) | 10 (6–16) | 4.8 (2.6–8.3) | 11 (8–17) | 6.3 (5.0–7.9) | 13 (8–21) | **<0.0001**^#^ | ns^#^ |
| **Granulocytes** | 43.0 (32.6–56.1) | 1921 (1275–2932) | 52.5 (34.3–63.7) | 2551 (1664–4212) | 48.1 (39.8–58.0) | 1874 (1240–2545) | ns^#^ | **0.0022**^#^ |
| **Cell Subsets** | **DwoWS (n=333)** | | **DwWS(n=231)** | | **SD (n=32)** | | **P value** | |
|  | ***%*** | ***Absolute count (/µl)*** | ***%*** | ***Absolute count (/µl)*** | ***%*** | ***Absolute count (/µl)*** | ***%*** | ***Absolute count (/µl)*** |
| **Lymphocytes** | 32.3 (22.2–39.3) | 1402 (823–2220) | 31.1 (21.8–39.7) | 1502 (952–2685) | 32.2 (19.5–38.6) | 1677 (995–2835) | ns^#^ | ns^#^ |
| **NK^++^ cells^a^** | 6.5 (4.3–9.2) | 87 (47–165) | 6.2 (4.0–8.6) | 94 (50–172) | 5.2 (2.8–9.6) | 100 (32–180) | ns^#^ | ns^#^ |
| **NK^+-^ cells^a^** | 1.1 (0.8–1.6) | 16 (9–29) | 1.2 (0.8–1.7) | 19 (10–33) | 1.3 (0.9–1.6) | 20 (14–33) | ns^#^ | ns^#^ |
| **NKT cells^a^** | 4.9 (3.8–7.5) | 75 (42–132) | 4.8 (3.7–7.1) | 78 (48–138) | 3.9 (3.2–6.0) | 80 (36–109) | ns^#^ | ns^#^ |
| **B cells^a^** | 9.9 (7.2–14) | 142 (72–262) | 10.9 (7.7–15.1) | 166 (339–87) | 12.6 (9.5–17.9) | 224 (107–405) | **0.0177**^#^ | **0.0144**^#^ |
| **T cells^a^** | 60.2 (53.9–65.7) | 769 (380–1264) | 59.2 (52.4–64.1) | 876 (472–1427) | 53.2 (44.9–59.6) | 803 (555–1380) | **<0.0001**^#^ | ns^#^ |
|  | **DwoWS (n=327)** | | **DwWS (n=227)** | | **SD (n=31)** | |  |  |
| **Monocytes** | 6.2 (4.4–8.5) | 283 (162–441) | 6.5 (4.8–8.4) | 318 (175–496) | 6.5 (5.1–7.6) | 372 (255–529) | ns^#^ | **0.0251**^#^ |
| **CM^b^** | 68.8 (60.4–76.1) | 207 (113–326) | 68 (60.6–76.5) | 241 (123–377) | 68.7 (62.5–76.2) | 268 (190–399) | ns^#^ | **0.0339**^#^ |
| **IM^b^** | 17.8 (12.6–24.5) | 49 (28–88) | 16.7 (12.6–24.8) | 56 (33–93) | 17.4 (10.3–24.1) | 57 (40–91) | ns^#^ | ns^#^ |
| **NCM^b^** | 2.1 (3.3–4.9) | 10 (5–15) | 3.2 (1.9–4.9) | 10 (6–17) | 2.9 (2.1–3.8) | 13 (7–17) | ns^#^ | ns^#^ |
| **Granulocytes** | 43 (32.7–56.7) | 1844 (1215–2706) | 43.5 (31.9–55.7) | 2019 (1290–3128) | 43.1 (35.2–51.9) | 2574 (1762–3160) | ns^#^ | **0.0184**^#^ |

The presented values are median (IQR).

^#^ - Kruskal-Wallis test followed by Bonferroni correction for multiple comparisons.

n=Number of subjects. % of cell subsets - (^a^) represents percentage of lymphocytes and (^b^) represents percentage of monocytes.

Abbreviations: FC – febrile controls; HC – healthy controls; ns - not significant; IQR – Inter Quartile Range; DwoWS – dengue without warning signs; DwWS – dengue with warning signs; SD –severe dengue; NKT – CD56^+^CD3^+^ natural killer T cells; NK^++^ - CD56^+^CD16^+^ natural killer cells; NK^+-^ - CD56^+^CD16^-^ natural killer cells; B cells – CD19^+^ cells; T cells – CD3^+^ cells; CM – CD14^+^CD16^-^ classical monocytes; IM – CD14^+^CD16^+^ intermediate monocytes; NCM – CD14^-^CD16^+^ non-classical monocytes.

**Supplementary Table 4.** **Percentage producers and MFI of cytokines from innate cell subsets.**

| **Cell Subsets** | **IFN-γ** | | **IP-10** | | **IL-10** | | | **TNF-α** | | |
| --- | --- | --- | --- | --- | --- | --- | --- | --- | --- | --- |
|  | ***% Producers***  ***[Median (IQR)]*** | ***MFI***  ***(AU x 10^3^)*** | ***% Producers***  ***[Median (IQR)]*** | ***MFI***  ***(AU x 10^3^)*** | ***% Producers***  ***[Median (IQR)]*** | | ***MFI***  ***(AU x 10^3^)*** | ***% Producers***  ***[Median (IQR)]*** | | ***MFI***  ***(AU x 10^3^)*** |
| **NKT** | 95.5  [1.5 (0.77-2.6)] | 2.2  (1.8-2.7) | 44.3  [0.06 (0.004-0.3)] | 6.1  (5.4-7.4) | 33.9  [0.04 (0.007-0.1)] | | 1.1  (0.8-1.5) | 98.2  [3.25 (2.0 -5.3)] | | 3.0  (2.3-4.2) |
| **NK++** | 81.9  [0.29 (0.16-0.56)] | 1.6  (1.3-1.9) | 48.3  [0.065(0.1-0.28)] | 5.5  (4.6-6.9) | 13.6  [0.009 (0-0.03)] | | 1.0  (0.8-1.5) | 95.1  [1.007 (0.57-1.61)] | | 1.5  (1.3-1.8) |
| **NK+-** | 13.8  [0.13 (0-0.33)] | 2.6  (1.3-4.2) | 21.0  [0.29 (0-0.29)] | 4.8  (0-6.9) | 1.5  [0 (0-0.03)] | | 0.7  (0-1.4) | 44.1  [0.52 (0.2-0.9)] | | 1.7  (1.0-3.1) |
|  | **IL-6** | | **IP-10** | | **IL-10** | | | **TNF-α** | | |
| **CM** | 28.2  [0.01 (0-0.14)] | 41.6  (30.3-57.4) | 47.0  [0.027 (0-0.13)] | 16.9  (12.9-22.9) | 35.6  [0.01 (0-0.05)] | 4.4  (3.6-5.7) | | 87.9  [0.2 (0.11-0.32)] | 2.8  (2.3-3.5) | |
| **IM** | 37.8  [0.31 (0-0.2)] | 89.2  (78.8-100.3) | 41.2  [0.05 (0-0.25)] | 52.2  (44.1-71.2) | 42.4  [0.02 (0-0.29)] | 6.5  (5.6-8.8) | | 88.7  [1.49 (0.5-3.1)] | 5.7  (4.4-7.4) | |
| **NCM** | 11.5  [0.54 (0-0.28)] | 22.1  (18.4-26.5) | 15.6  [0.1 (0-0.42)] | 10.9  (9.6-13.5) | 19.8  [0.09 (0-0.44)] | 1.8  (1.5-2.3) | | 98.8  [40.6 (24.6-59)] | 3.3  (2.2-5.3) | |
| **Granulocytes** | 76.8  [0.015 (0.005-0.03)] | 60.5  (48.1-88.2) | 74.4  [0.019 (0.005-0.06)] | 38.7  (30.0-52.8) | 73.3  [0.025 (0.003-0.05)] | 6.8  (5.2-8.6) | | 98.8  [0.414 (0.20-0.71)] | 3.7  (3.6-4.4) | |

% producers describe the percentage of patients in our cohort with detectable cytokine events from each cell subset; median cytokine secreting cells as a percentage of parent and IQR are represented within brackets.

The tabulated MFI (AU x 10^3^) are median (IQR).

Abbreviations: MFI - Median Fluorescence Intensity; AU – Arbitrary Unit; IQR – Inter Quartile Range; NKT – CD56^+^CD3^+^ natural killer T cells; NK^++^ - CD56^+^CD16^+^ natural killer cells; NK^+-^ - CD56^+^CD16^-^ natural killer cells; CM – CD14^+^ CD16^-^ classical monocytes; IM – CD14^+^CD16^+^ intermediate monocytes; NCM – CD14^-^CD16^+^ non-classical monocytes.
