# Supplementary figures and images for: Innate Immune Cytokine Profiling and Biomarker Identification for Outcome in Dengue Patients
Source: Front Immunol. 2021 Jul 14;12:677874. doi: 10.3389/fimmu.2021.677874 (PMC8318829; doi:10.3389/fimmu.2021.677874)

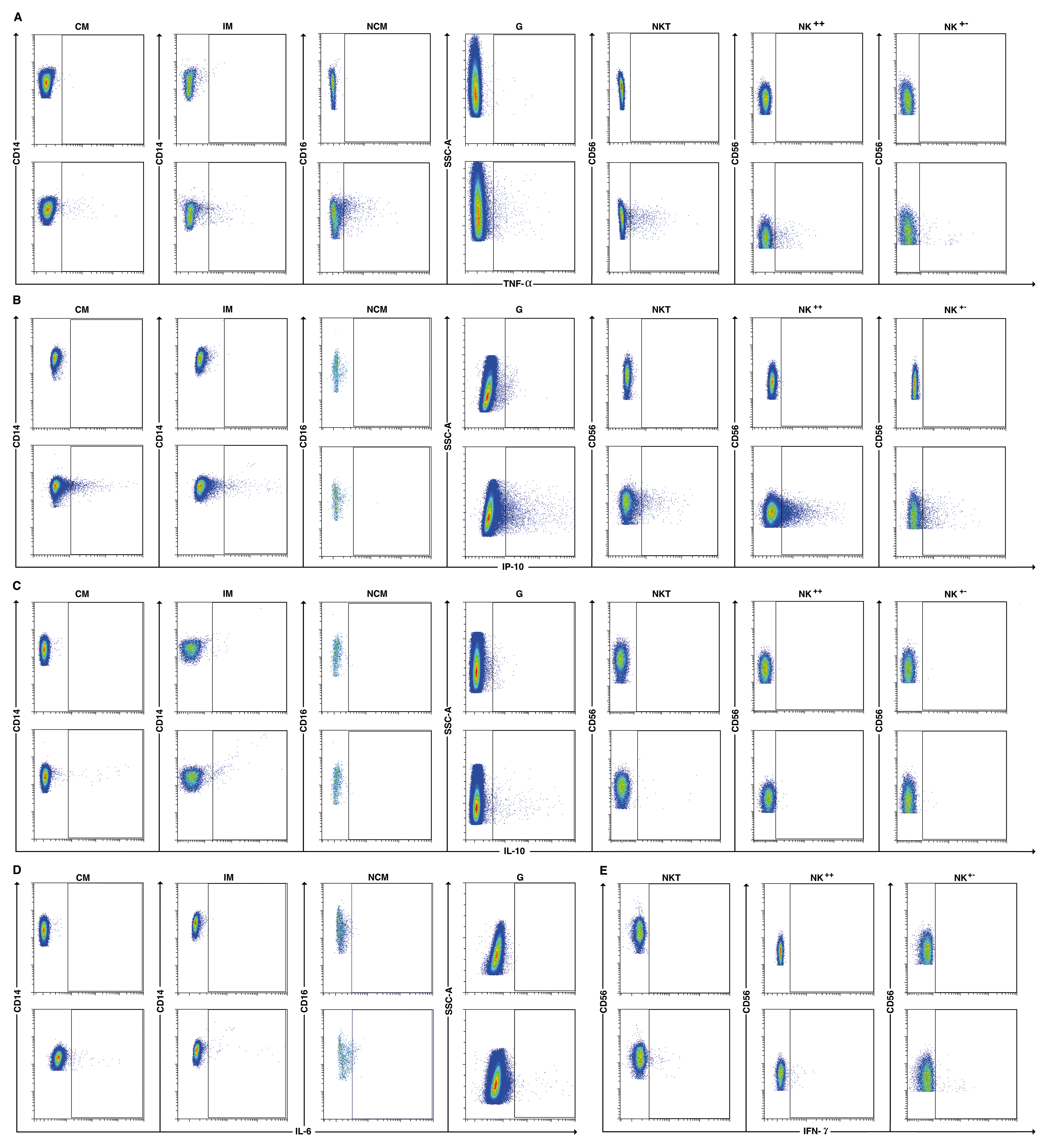

Supplement: Supplementary file 2 [file Image_1.tif]

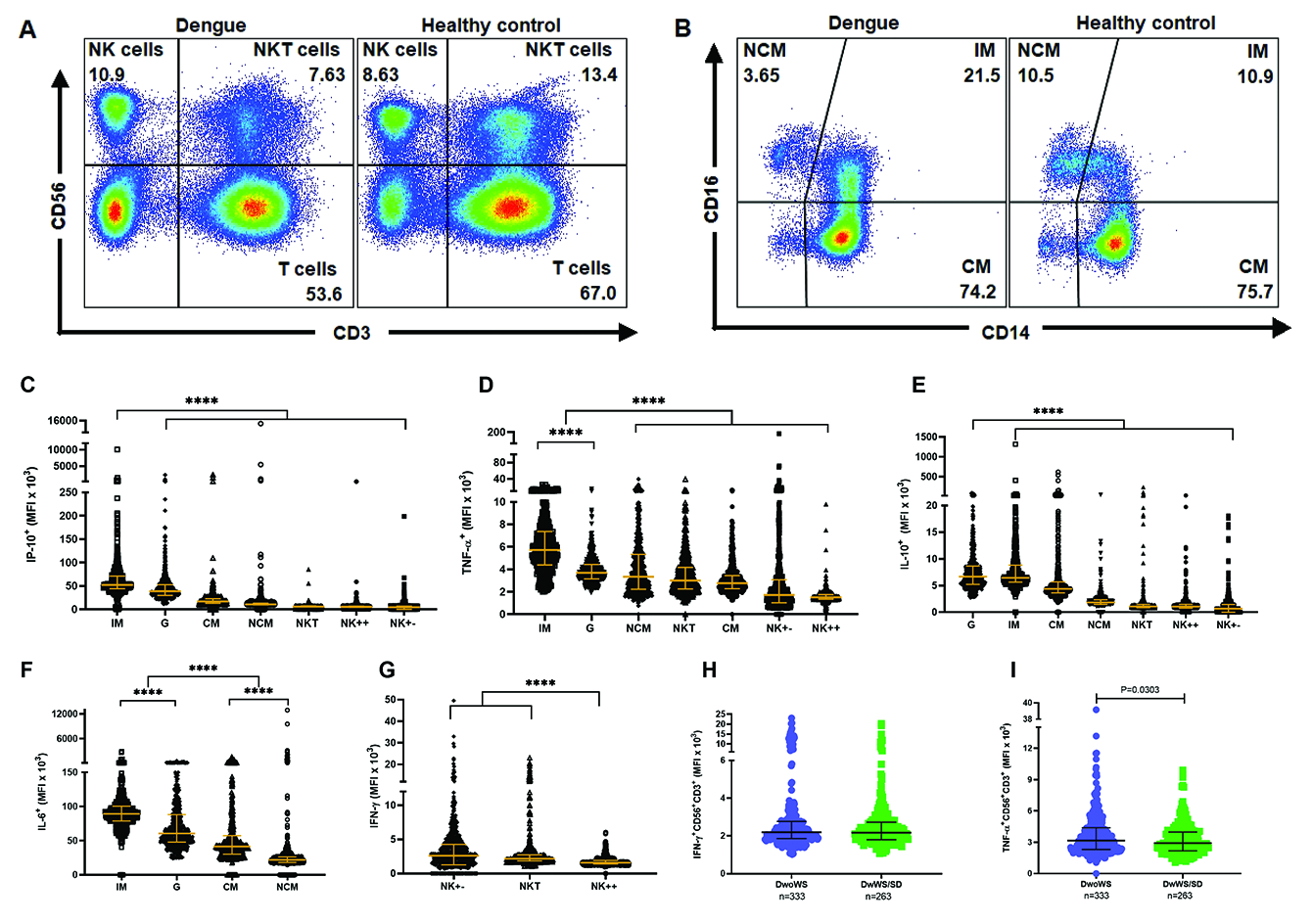

Supplement: Supplementary file 3 [file Image_2.tif]
